# Association between preoperative anxiety and ciprofol requirements in women undergoing surgical abortion: a prospective observational study

**DOI:** 10.3389/fmed.2025.1706386

**Published:** 2025-12-19

**Authors:** Baozhu Cha, Zhonghao Shao, Junchao Lv, Jiafei Cao, Qianwen Qin

**Affiliations:** Department of Anesthesiology, Affiliated Hospital of Shaoxing University, Shaoxing, Zhejiang, China

**Keywords:** ciprofol, anxiety, STAI, alfentanil, abortion

## Abstract

**Objective:**

To investigate the association between preoperative anxiety level and ciprofol consumption in patients undergoing surgical abortion.

**Methods:**

A total of 144 patients scheduled for surgical abortion were enrolled and completed both the State–Trait Anxiety Inventory (STAI) and the Self-Rating Anxiety Scale (SAS). Anesthesia was induced with 5 μg/kg alfentanil and 0.3 mg/kg ciprofol. Additional ciprofol (0.1 mg/kg each) was administered as needed to maintain adequate anesthetic depth. The primary outcomes were the total ciprofol dose and State Anxiety (S-STAI) score. Secondary outcomes included the Trait Anxiety (T-STAI) and SAS scores, induction time, awakening time, baseline hemodynamic profiles, and the incidence of adverse events.

**Results:**

Preoperative anxiety levels (S-STAI) were weakly and positively associated with total ciprofol dose (*r_s_* = 0.216, 95%*CI* [0.049, 0.371], *p* = 0.009), induction time (*r_s_* = 0.312, *95%CI* [0.1521, 0.4567]*, p* < 0.001), baseline heart rate (HR) (*r_s_* = 0.321, 95%*CI* [0.1611, 0.4640], *p* < 0.001), and mean arterial pressure (MAP) (*r_s_* = 0.213, 95%*CI* [0.04579, 0.3676], *p* = 0.011). No significant association was observed between preoperative anxiety and awakening time (*r_s_* = −0.156, 95%*CI* [−0.3163, 0.01239], *p* = 0.062). Furthermore, after multivariate linear regression analysis considering confounding factors, preoperative anxiety remained a statistically significant, though modest, independent variable associated with total ciprofol dose (β = 0.187, 95%*CI* [0.089, 0.285]). Subgroup analysis showed a significant overall difference in total ciprofol dose (*p* = 0.032) and baseline MAP (*p* = 0.044) across anxiety groups, although pairwise comparisons were not statistically significant. Baseline HR was higher in both moderate and severe anxiety groups, while induction time was longer only in the moderate-anxiety group, compared with the mild-anxiety group. The incidence of adverse events did not differ significantly among the groups (all *p* > 0.05).

**Conclusion:**

Preoperative anxiety showed a modest association with ciprofol requirements and induction time. These results indicate that anxiety may be a relevant factor to consider during sedation management for surgical abortion.

**Clinical trial registration:**

ChiCTR2500108366 (Chinese Clinical Trial Registry, www.chictr.org.cn).

## 1 Introduction

Surgical abortion broadly refers to procedural methods for terminating unintended pregnancies. In this study, the term specifically refers to first-trimester vacuum aspiration, the standard technique recommended for pregnancies up to 14 weeks (1). Globally, between 2015 and 2019, there were approximately 121 million unintended pregnancies annually, with about 61% (roughly 73 million) ending in abortion (2). In China, about 9.8 million women undergo abortion annually (3). Despite improvements in abortion techniques, physical and psychological discomfort remains common.

Pain is one of the most common experiences during surgical abortion, with approximately 78 to 97% of patients reporting moderate to severe intraoperative pain, often accompanied by nausea, dizziness, sweating, and blood pressure fluctuations, which significantly affects procedural experience and treatment adherence (4). While mild to moderate pain is an expected part of the procedure, severe or prolonged pain beyond typical levels may indicate complications and should be evaluated clinically. To alleviate intraoperative discomfort, sedatives and analgesics are commonly administered in combination to facilitate the procedure (5). However, intraoperative experiences are influenced not only by pharmacological interventions but also by the patient’s preoperative psychological state. Studies have shown that more than 60% of patients experience varying degrees of anxiety before surgery (6). Preoperative anxiety may alter sedative requirements, exacerbate hemodynamic fluctuations, and increase the risk of adverse events, thereby complicating intraoperative management and postoperative recovery (7).

Ciprofol is a newly developed short-acting intravenous sedative with rapid onset, fast metabolism, minimal accumulation, and mild cardiovascular and respiratory effects (8, 9). Although its pharmacodynamic profile has been studied in minor outpatient procedures (10, 11), the association between preoperative anxiety and ciprofol requirements remains unclear, particularly in patients undergoing surgical abortion. Most existing evidence on anxiety and sedative requirements has focused on propofol (12, 13), resulting in an important knowledge gap regarding ciprofol in this patient population.

Understanding the effect of preoperative anxiety on ciprofol consumption may provide valuable insights for perioperative management and support the development of individualized sedation strategies. Therefore, this prospective study aimed to examine the association between preoperative anxiety levels and ciprofol requirements in patients undergoing surgical abortion.

## 2 Methods

### 2.1 Patients

A total of 144 patients who underwent first-trimester surgical abortion (vacuum aspiration) at the Affiliated Hospital of Shaoxing University from February 2025 to September 2025 were enrolled in this study. Before each study participant enrollment, this study was approved by the hospital’s ethics committee [2024(Yan)-045-01] and was registered in the Chinese Clinical Trials Registry (ChiCTR2500108366, 2025/08/28). Every individual provided informed consent in writing.

### 2.2 Inclusion and exclusion criteria

Inclusion criteria were: female patients aged 18–45 years; body mass index (BMI), 18–28 kg/m^2^; American Society of Anesthesiologists (ASA) class I-II; no coagulation disorder.

Exclusion criteria were: known allergy to the study anesthetic drugs; major systemic disorders such as hepatic or renal diseases; communication or respiratory difficulties, including recent respiratory infections; and a clinical diagnosis of anxiety or depression with active pharmacological treatment (i.e., documented use of anxiolytics, antidepressants, or other sedative medications).

### 2.3 Blinding and data collection

Anxiety levels were assessed in the preoperative room prior to the induction of anaesthesia using the State–Trait Anxiety Inventory (STAI) and the Self-Rating Anxiety Scale (SAS). Thirty to sixty minutes before surgery, an anesthesiologist collected patients’ demographic and medical history data and completed the anxiety assessments. Subsequently, another anesthesiologist who was not involved in data collection was responsible for anesthesia induction and maintenance. Intraoperative and postoperative data were collected separately by an anesthesiologist and an anesthesia nurse, both of whom were blinded to the patients’ preoperative anxiety scores.

### 2.4 Evaluation of anxiety

In this study, preoperative anxiety was assessed using the State–Trait Anxiety Inventory (STAI) and the Self-Rating Anxiety Scale (SAS), with the STAI considered the gold standard (14). The STAI comprises two independent subscales: State Anxiety (S-STAI) and Trait Anxiety (T-STAI). State Anxiety evaluates the patient’s current situational tension before surgery, whereas Trait Anxiety measures stable, anxiety-prone personality traits. Each STAI subscale contains 20 items, totaling 40 items, with scores ranging from 20 to 80; higher scores indicate greater anxiety severity. For both S-STAI and T-STAI, patients were classified into three categories: mild (20–37), moderate (38–44), and severe anxiety (45–80) (15).

The SAS was simultaneously used to assess the frequency and severity of anxiety symptoms experienced by patients during the week preceding surgery (16). The scale comprises 20 items, with higher total scores indicating greater levels of anxiety. Detailed descriptions of the STAI and SAS used in this study are provided in Supplementary Files 1 and 2, respectively.

### 2.5 Study protocol

Participants fasted for at least 8 h and abstained from drinking for 2 h before the procedure; no premedication was administered. Anxiety was assessed upon arrival at the preoperative waiting room. Upon arrival at the operating room, standard monitoring procedures were established, including electrocardiography (ECG), non-invasive blood pressure [including systolic blood pressure (SBP) and diastolic blood pressure (DBP)], HR, pulse oximetry (SpO2), and respiratory rate (RR). Oxygen was administered at 5 L/min via a nasal cannula. A 22-gauge cannula was inserted into the patient’s hand vein. Ringer’s lactate solution (5 mL/kg/h) was infused to maintain patency. All patients received ciprofol (0.3 mg/kg) and alfentanil (5 μg/kg) for induction. The Modified Observer’s Alertness/Sedation scale (MOAA/S) was assessed every 20 s after administration; surgery commenced only if the MOAA/S score was ≤ 1 at 120 s. All surgical procedures were performed by two experienced surgeons following a standardized protocol. If intraoperative body movement occurred or the MOAA/S score was ≥2, an additional dose of ciprofol (0.1 mg/kg) was administered as needed until the completion of surgery. The number of supplemental ciprofol boluses was recorded. In cases of adverse events, norepinephrine (20–40 μg) was administered to patients with hypotension. In patients with respiratory depression, the jaw was lifted to open the airway and oxygen flow was increased using a pressurized mask; endotracheal intubation was performed if this was ineffective.

### 2.6 Outcomes

#### 2.6.1 Primary outcomes

The total ciprofol dose and the S-STAI score.

#### 2.6.2 Secondary outcomes

Secondary outcomes included SAS and T-STAI scores, baseline hemodynamics, induction time, awakening time, and adverse events.

The baseline hemodynamic profiles {SBP, DBP, MAP [MAP = (SBP + 2 × DBP)/3]}, HR, and SpO2 were recorded upon the patient’s arrival in the operating room. Induction time was defined as the interval from ciprofol administration to the patient achieving an MOAA/S score ≤1. Awakening time was defined as the interval from the last ciprofol dose to full alertness (MOAA/S = 5).

All adverse events during the study period were documented, including injection pain, hypotension, and hypoxemia. Injection pain referred to the pain reported verbally by patients after the first injection. Hypotension was considered a ≥ 30% reduction in MAP from baseline. Hypoxemia was defined as SpO2 below 90% during the study (17).

### 2.7 Sample size calculation

Based on previous research (*n* = 60), the correlation between total ciprofol dose and SAS (r_s_ = 0.258, *p* = 0.047) and STAI-S (r_s_ = 0.343, *p* = 0.007), this study adopted a conservative principle by selecting the weaker correlation effect (*r_s_* = 0.258) for sample size estimation. Sample size estimation was performed using the “Correlation” module of PASS 15.0 software (NCSS, LLC): α = 0.05, 1 − β = 0.8. 115 participants were needed, anticipating a 20% dropout rate, we enrolled 144 participants in the study.

### 2.8 Statistical analysis

All statistical analyses were performed using SPSS 20.0 (IBM Corp., Chicago, IL, USA) and GraphPad Prism 9.5 (GraphPad Software, San Diego, CA, USA). Normality of continuous variables was assessed using the Shapiro–Wilk test. Normally distributed data were expressed as mean ± standard deviation (SD) and compared using one-way analysis of variance (ANOVA) with Bonferroni *post hoc* tests. Non-normally distributed data were presented as median (interquartile range, IQR) and analyzed using the Kruskal Wallis test, followed by Dunn’s post hoc test with Bonferroni correction. Spearman’s correlation coefficient was applied to assess linear relationships between continuous variables. Categorical variables were presented as n (%) and compared using the chi-squared test or Fisher’s exact test, as appropriate. Effect sizes for group comparisons were quantified using eta-squared (η^2^), calculated from ANOVA sums of squares as:


$$\eta^{2}=\frac{SS_{\text{between}}}{SS_{\text{total}}}$$

and further expressed as Cohen’s f using:


$$f=\sqrt{\frac{\eta^{2}}{1-\eta^{2}}}$$

A multivariable linear regression analysis was performed to evaluate the robustness of the results. Statistical significance was defined as *p* < 0.05.

## 3 Results

### 3.1 Patient demographic characteristics

**Figure 1:**
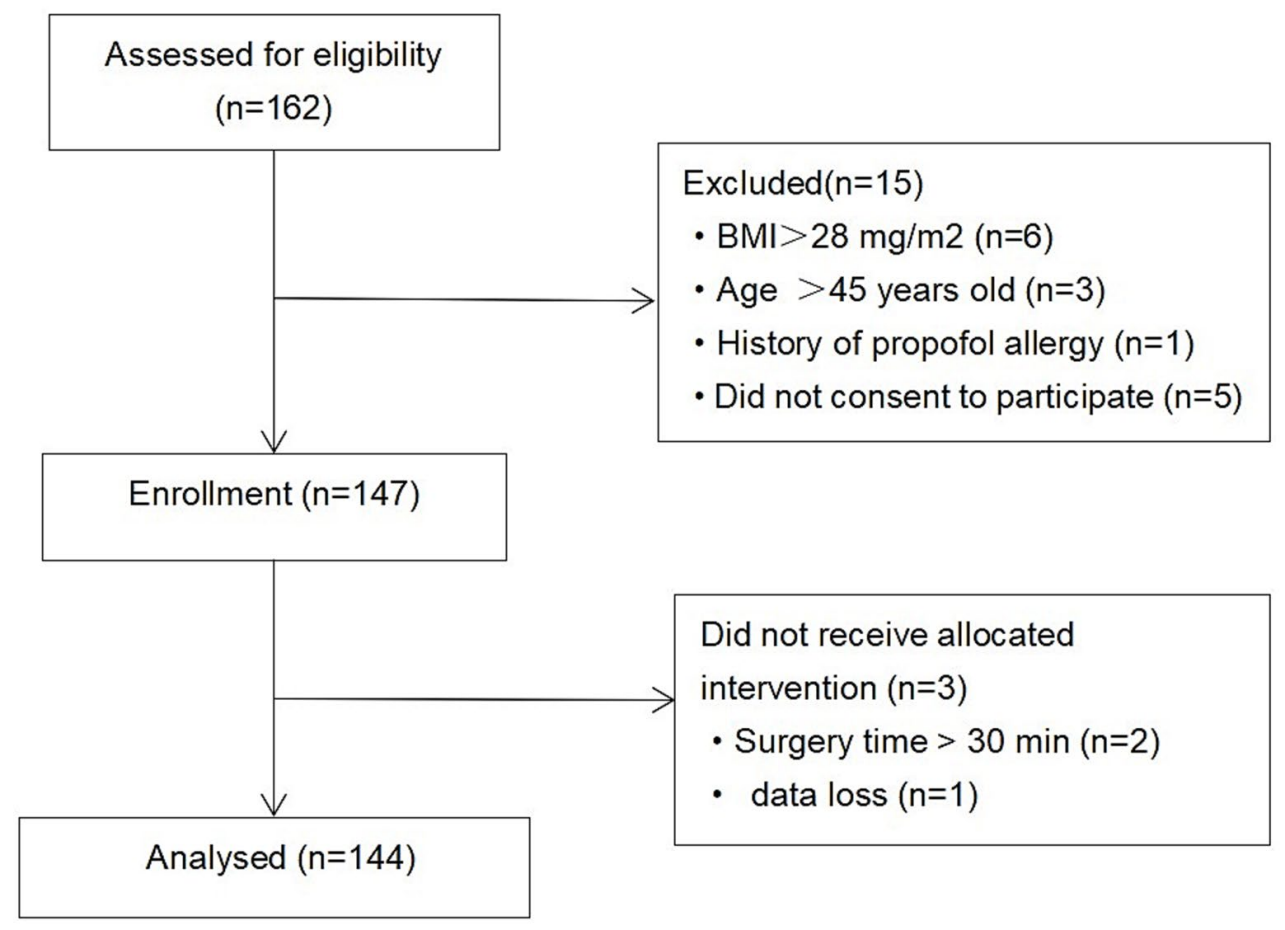
Study enrollment flow diagram.

This study flow diagram is shown in Figure 1. A total of 162 patients were enrolled, of whom 15 were excluded for the following reasons: BMI > 28 kg/m^2^ (*n* = 6), age> 45 (*n* = 3), allergies to propofol (*n* = 1), or refusal to provide informed consent (*n* = 5). During the study, three additional participants were excluded from follow-up and analysis owing to prolonged surgery duration (> 30 min; *n* = 2) or loss of primary outcome data (*n* = 1). Ultimately, 144 patients were included in the final analysis. The patients’ characteristics and baseline hemodynamic profiles are shown in Table 1.

**Table 1:** Baseline characteristics of patients.

Characteristics	
Age (years)	35.00 (31.00, 38.00)
Weight (kg)	58.48 ± 8.00
Height (cm)	160.00 (157.00, 163.00)
BMI (kg/m^2^)	22.74 ± 2.87
ASA (I/II)	131/13
Education level (Primary school/Junior high school/High school/University or higher)	6/32/48/58
Gestational age (weeks)	6.71 (6.00, 7.57)
Baseline hemodynamic profiles	
Baseline SBP (mmHg)	122.00 (113.00, 131.00)
Baseline DBP (mmHg)	74.00 (67.25, 81.00)
Baseline MBP (mmHg)	89.67 (84.00, 96.56)
Baseline HR (beat/min)	74.00 (67.00, 84.75)

Data are expressed as means ± SD, or median (IQR). BMI, body mass index; ASA, American Society of Anesthesiologists; SBP, systolic blood pressure; DBP, diastolic blood pressure; MAP, mean arterial pressure; HR, heart rate.

### 3.2 Primary outcomes

**Figure 2:**
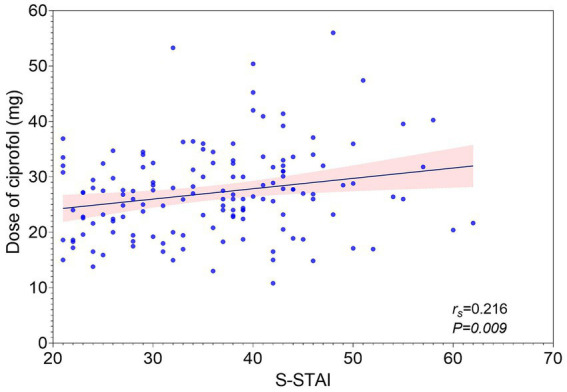
Correlation between State Anxiety (S-STAI) and ciprofol total dose.

A weak but statistically significant correlation was observed between the total ciprofol dose and the S-STAI score (*r_s_* = 0.216, 95%*CI* [0.049, 0.371], *p* = 0.009), as shown in Figure 2.

### 3.3 Secondary outcomes

**Figure 3:**
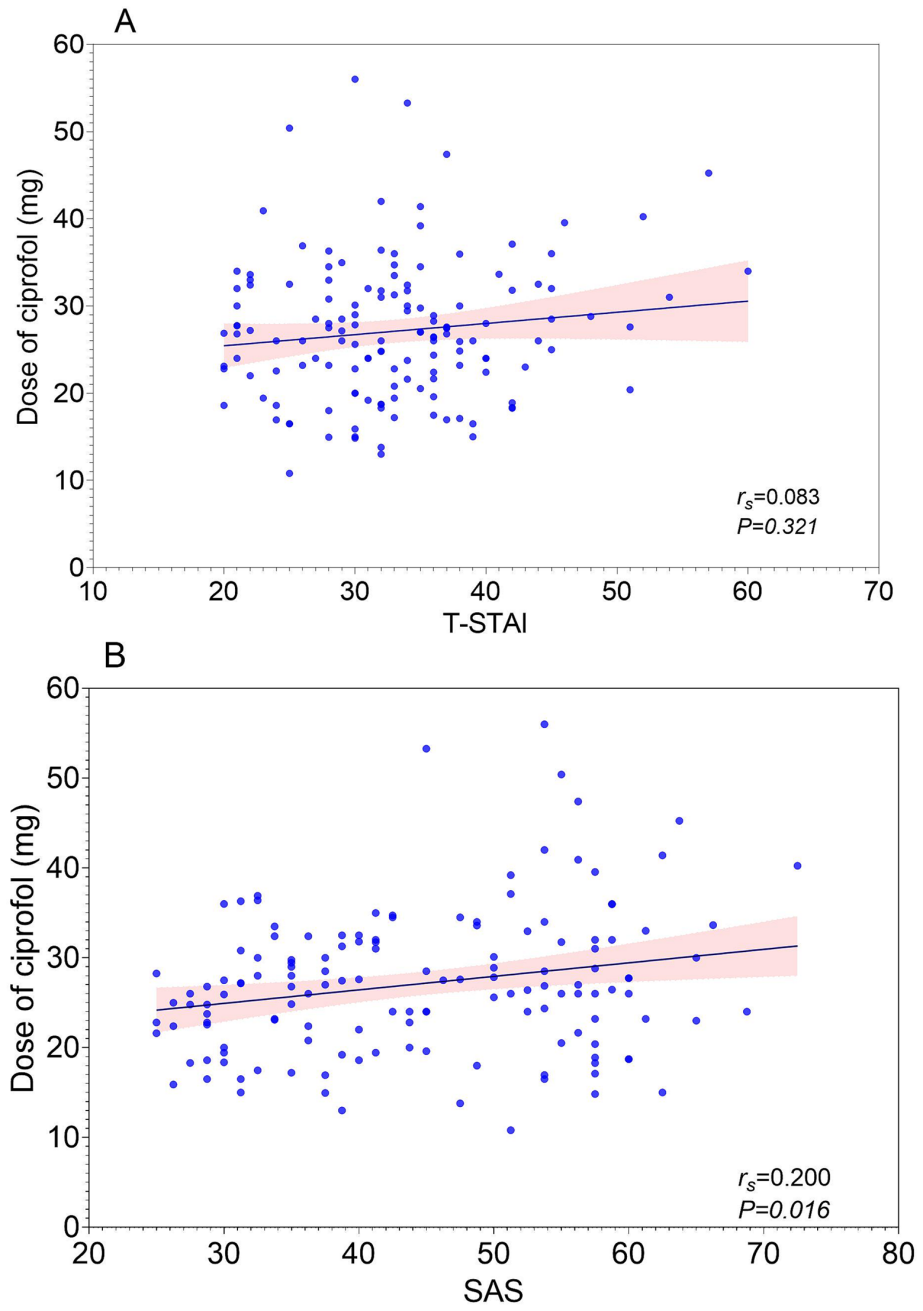
**(A)** Correlation between Trait Anxiety (T-STAI) and ciprofol total dose. **(B)** Correlation between SAS and ciprofol total dose.

**Figure 4:**
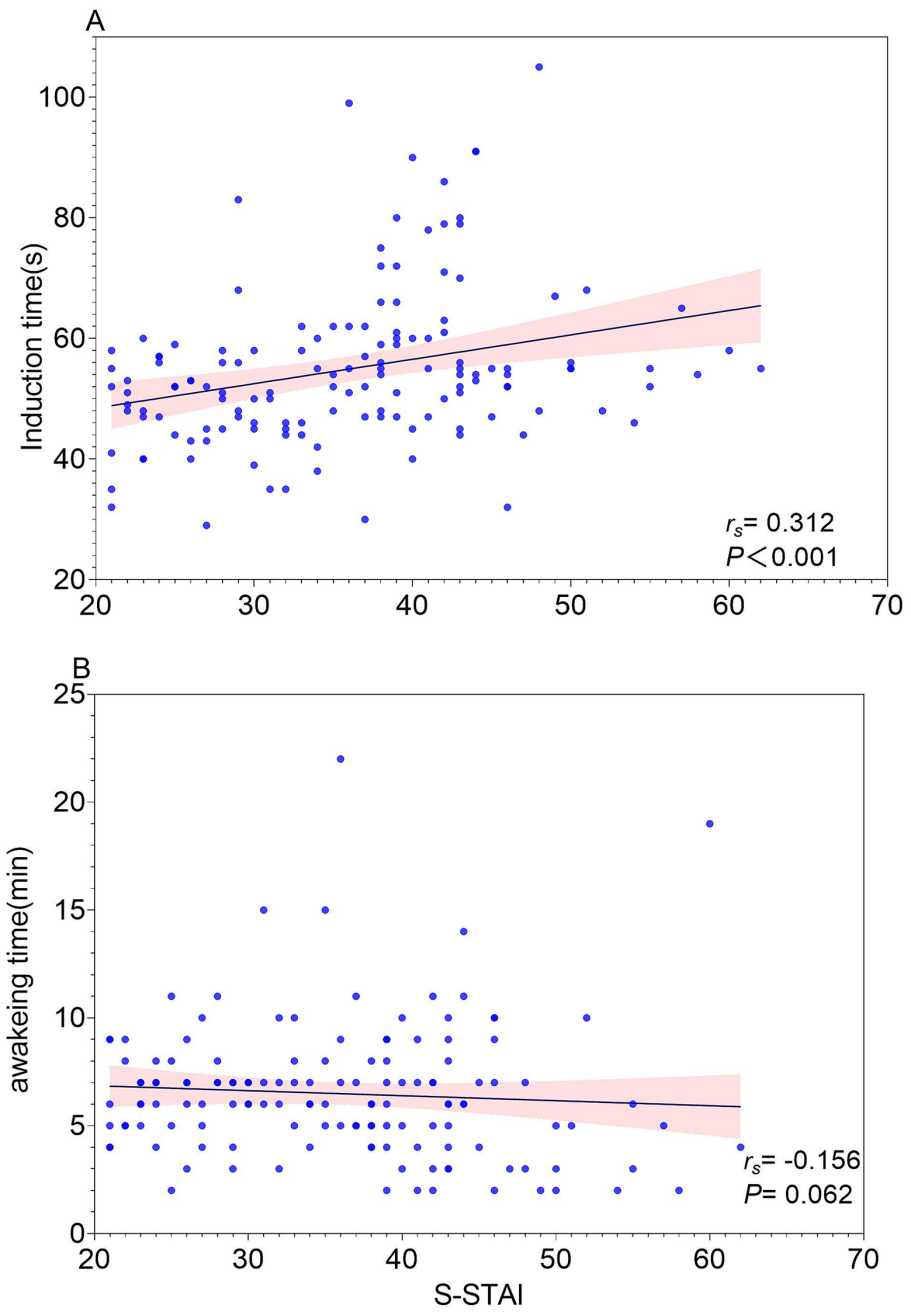
**(A)** Correlation between State Anxiety (S-STAI) and induction time. **(B)** Correlation between State Anxiety (S-STAI) score and awakeing time.

**Figure 5:**
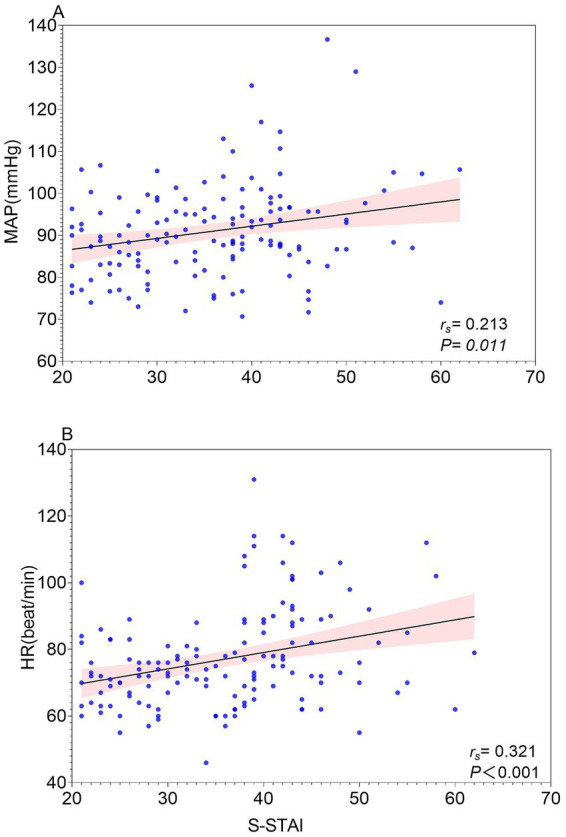
**(A)** Correlation between State Anxiety (S-STAI) and mean arterial pressure (MAP). **(B)** Correlation between State Anxiety (S-STAI) and baseline Heart Rate (HR).

Results for the secondary outcomes are presented in Table 2, Figures 3–5. Spearman’s correlation analysis showed that S-STAI demonstrated a modest positive association with induction time (*r_s_* = 0.312, 95%*CI* [0.1521, 0.4567], *p* < 0.001), baseline heart rate (HR) (*r_s_* = 0.321, 95%*CI* [0.1611, 0.4640], *p* < 0.001), and mean arterial pressure (MAP) (*r_s_* = 0.213, 95%*CI* [0.04579, 0.3676], *p* = 0.011). No significant correlation was observed between S-STAI scores and awakening time (*r_s_* = −0.156, 95%*CI* [−0.3163, 0.01239], *p* = 0.062).

**Table 2:** Level of anxiety and sedation results.

Pre-procedural anxiety	
S-STAI	37.00 (28.00,43.00)
T-STAI	32.50 (28.00,37.00)
SAS	43.75 (33.75, 55.00)
Total ciprofol dose (mg)	26.43 (18.86, 31.46)
Operative duration (min)	12.00 (8.75, 17.25)
Induction time (s)	52.00 (46.75, 58.00)
Awaking time (min)	6.00 (5.00, 8.00)

Values are expressed as the medians (IQR). S-STAI, state anxiety; T-STAI, trait anxiety; SAS, self-rating anxiety scale.

T-STAI was not significantly correlated with total ciprofol dose (*r_s_* = 0.083, 95% *CI* [−0.08617, 0.2482], *p* = 0.360), whereas SAS showed a weak positive correlation with total ciprofol dose (*r_s_* = 0.200, 95% *CI* [0.033, 0.356], *p* = 0.016).

### 3.4 Multivariable linear regression analysis

A multivariable linear regression model was used to examine the association between preoperative anxiety and total ciprofol dose, adjusting for operative duration, gestational age, age, and BMI. The analysis showed that S-STAI (β = 0.187, 95% *CI* [0.089, 0.285], *p* < 0.001), operative duration (β = 0.935, 95% *CI* [0.736, 1.134], *p* < 0.001), age (β = −0.212, 95% *CI* [−0.368, −0.055], *p* = 0.008), and BMI (β = 1.070, 95% *CI* [0.749, 1.392], *p* < 0.001) were independently associated with total ciprofol dose. In contrast, gestational age was not significantly associated with total dose (β = −0.031, 95% *CI* [−0.624, 0.561], *p* = 0.917) (Table 3).

**Table 3:** Multivariable linear regression for total ciprofol dose.

Independent variable	*p*	Unstandardized coefficients B	95% *CI* for B
(Constant)	0.161	−7.824	−18.805 ~ 3.158
S-STAI	<0.001	0.187	0.089 ~ 0.285
Operative duration (min)	<0.001	0.935	0.736 ~ 1.134
Gestational age (weeks)	0.917	−0.031	−0.624 ~ 0.561
Age (years)	0.008	−0.212	−0.368 ~ −0.055
BMI (kg/m^2^)	<0.001	1.070	0.749 ~ 1.392

S-STAI, state anxiety; BMI, body mass index; CI, confidence interval.

### 3.5 Subgroup analysis

According to S-STAI scores, patients were classified into mild (*n* = 76), moderate (*n* = 43), and severe anxiety groups (Table 4). Total ciprofol dose differed significantly at the overall level (*H* = 3.53, *p* = 0.032), with a small effect size (η^2^ = 0.048; Cohen’s *f* = 0.225), although no pairwise comparisons were significant.

**Table 4:** Subgroup analysis.

Variable	Mild anxiety group (*n* = 76)	Moderate anxiety group (*n* = 43)	Severe anxiety group (*n* = 25)	*F*/χ^2^/*H*	*P*	η^2^	Cohen’s f/v
Total ciprofol dose (mg)	24.92 (19.48, 29.94)	28.78 ± 8.01	29.20 ± 9.70	3.53	0.032	0.048	0.225
Induction time (s)	51.00 (45.00, 56.00)	60.00 (52.00, 72.00)*	54.00 (48.00, 55.50)	21.24	<0.001	0.136	-
Baseline HR (beat/min)	70.64 ± 9.26	86.40 ± 16.74*	80.72 ± 14.99*	21.55	<0.001	0.234	0.553
Baseline MBP (mmHg)	88.88 ± 8.98	93.91 ± 10.77	88.33 (83.17, 99.17)	3.19	0.044	0.043	0.213
Injection pain	0 (0)	0 (0)	0 (0)	-	>0.99	*-*	*-*
Hypotension	3 (3.95)	2 (4.65)	4 (16)	4.18	0.105	-	0.120
Hypoxemia	4 (5.26)	2 (4.65)	2 (8.00)	0.64	0.783	-	0.047

Values are expressed as the means ± SD, medians (IQR), or number (percentage) of patients. *p < 0.05, cf. Mild anxiety group.

Induction time also differed among groups (*H* = 21.24, *p* < 0.001; η^2^ = 0.136); post-hoc testing showed a longer induction time in the moderate- versus mild-anxiety group (*p* < 0.05), with no other significant contrasts. Baseline HR showed a significant group effect (*F* = 21.55, *p* < 0.001; η^2^ = 0.234; Cohen’s *f* = 0.553), with both moderate- and severe-anxiety groups exhibiting higher HR than the mild-anxiety group (p < 0.05). Baseline MAP differed at the global level (*F* = 3.19, *p* = 0.044; η^2^ = 0.043; Cohen’s *f* = 0.213), but no pairwise comparisons reached significance.

The incidence of adverse events did not differ among groups. Cramer’s V was 0.120 for hypotension and 0.047 for hypoxemia (all *p* > 0.05). Injection pain was absent in all groups.

## 4 Discussion

This prospective observational study found that higher preoperative anxiety, as assessed by S-STAI, was modestly but significantly associated with increased ciprofol consumption and prolonged induction time in women undergoing surgical abortion. The association remained significant after adjustment for relevant clinical factors, indicating that anxiety contributes independently to variability in sedative requirements. Patients with higher anxiety also exhibited elevated baseline heart rates, whereas awakening time and adverse events were comparable across anxiety levels.

The clinical relevance of this association becomes clearer when considering the procedural characteristics of surgical abortion (18). Although the procedure is brief, it is physiologically and emotionally stimulating, and patients with higher preoperative anxiety tend to exhibit increased autonomic arousal. Such heightened sympathetic activity may influence the pharmacodynamic response to sedatives, leading to greater overall sedative requirements throughout the procedure. Recognizing anxiety-related variability in drug demand can help clinicians anticipate dosing needs and ensure patient comfort and procedural safety.

Previous studies with propofol in endoscopic and surgical procedures have consistently shown that preoperative anxiety increases sedative requirements and exacerbates hemodynamic fluctuations (19–21). Our findings extend these observations to ciprofol, a newer GABAergic sedative with rapid metabolism and favorable cardiovascular stability, specifically in the abortion-surgery setting (22). Regression analyses further indicated that preoperative anxiety was independently associated with ciprofol dosing. Consistent with prior knowledge, state anxiety (S-STAI), reflecting acute emotional arousal, demonstrated stronger associations with anesthetic responses than trait anxiety (T-STAI) (23, 24). This is consistent with the concept that the immediate preoperative emotional state may contribute to variations in anesthetic requirements.

In the subgroup analysis, although a statistically significant overall difference in total ciprofol consumption was observed, post-hoc tests revealed no significant pairwise comparisons. The small effect size (η^2^ = 0.048) indicated substantial overlap in dosing distributions across groups. This pattern likely resulted from the combination of a limited effect magnitude, unequal group sizes, and reduced statistical power after multiple-testing correction (25). In particular, the severe-anxiety subgroup had a relatively small sample size, which further limited the power to detect pairwise differences. However, a more consistent and clinically interpretable gradient was observed for physiological parameters: both induction time and baseline heart rate showed clearer separations, being significantly higher in the moderate anxiety group relative to the mild group. This suggests that, in our study, these objective physiological markers may serve as more sensitive indicators of a patient’s anxious state than the absolute sedative dose recorded.

Although preoperative anxiety was associated with a prolonged induction time, awakening times and adverse event rates were comparable across anxiety groups. This discrepancy can be explained by ciprofol’s pharmacodynamic profile: the sympathetic activation associated with anxiety may delay the achievement of effective hypnotic drug levels in the central nervous system (26–28), which may be correlated with an increased sedative demand in our study. Once adequate sedation was achieved, the drug’s rapid metabolism predominantly determines its elimination, which resulted in consistent recovery profiles. The similar incidence of adverse events across anxiety levels further supports the safety and stability of ciprofol within the dosing range required.

From a mechanistic perspective, the observed associations may be linked to neurophysiological changes induced by preoperative anxiety. Activation of the sympathetic-adrenal system and hypothalamic–pituitary–adrenal axis increases circulating catecholamines and cortisol (29, 30), which could heighten central nervous system arousal and elevate the anesthetic threshold. Preclinical studies further suggest that anxiety-like states can reduce GABAA receptor sensitivity (31, 32), potentially diminishing the efficacy of GABAergic sedatives like ciprofol. While these mechanisms provide a plausible biological framework for our findings, they remain speculative in the clinical context and warrant further investigation.

This study has several limitations. First, the single-center design and moderate sample size, particularly the small subgroup of patients with severe anxiety (*n* = 25), limited the statistical power for detailed pairwise comparisons in the subgroup analysis. Second, restricting enrollment to relatively healthy women (ASA I–II) undergoing first-trimester surgical abortion may limit generalizability of our findings. Third, the absence of a propofol comparator precludes determining whether the observed anxiety–sedation relationship is specific to ciprofol or extends to other GABAergic sedatives. Finally, anxiety was assessed solely with validated questionnaires, without objective biomarkers or neurophysiological measures, which could have offered a more comprehensive evaluation of preoperative stress responses.

## 5 Conclusion

In summary, preoperative anxiety showed a weak but statistically significant association with higher total ciprofol requirements and slower induction in women undergoing surgical abortion, while awakening time and perioperative adverse events were unaffected. These findings suggest that anxiety may contribute to variability in overall sedative requirements. Future research should determine whether routine preoperative anxiety assessment could help guide individualized dosing strategies in this setting.

## Data Availability

The original contributions presented in the study are included in the article/Supplementary material, further inquiries can be directed to the corresponding author.
