# Supplementary material for: Association between preoperative anxiety and ciprofol requirements in women undergoing surgical abortion: a prospective observational study
Source: Front Med (Lausanne). 2025 Dec 19;12:1706386. doi: 10.3389/fmed.2025.1706386 (PMC12757401; doi:10.3389/fmed.2025.1706386)
Supplement: Supplementary file 1 [file Data_Sheet_1.pdf]

Table 1. STAI – State Anxiety Scale

NO.	Statement	1	2	3	4
*1	I feel calm				
*2	I feel secure				
3	I am tense				
4	I feel strained				
*5	I feel at ease				
6	I feel upset				
7	I am presently worrying over possible misfortunes				
*8	I feel satisfied				
9	I feel frightened				
*10	I feel comfortable				
*11	I feel self-confident				
12	I feel nervous				
13	I am jittery				
14	I feel indecisive				
*15	I am relaxed				
*16	I feel content				
17	I am worried				
18	I feel confused				
*19	I feel steady				
*20	I feel pleasant				

Table 2. STAI – Trait Anxiety Scale

NO.	Statement	1	2	3	4
*21	I feel pleasant				
22	I feel nervous and restless				
*23	I feel satisfied with myself				
*24	I wish I could be as happy as others seem to be				
25	I feel like a failure				

*26	I feel rested				
*27	I am “calm, cool, and collected”				
28	I feel that difficulties are piling up so that I cannot overcome them				
29	I worry too much over something that really doesn’t matter				
*30	I am happy.				
31	I have disturbing thoughts.				
32	I lack self-confidence				
*33	I feel secure				
*34	I make decisions easily				
35	I feel inadequate				
*36	I am content				
37	Some unimportant thought runs through my mind and bothers me				
38	I take disappointments so keenly that I can’t put them out of my mind				
*39	I am a steady person				
40	I get in a state of tension or turmoil as I think over my recent concerns				

Scoring Method:

Items 1–20 constitute the State Anxiety Inventory, which primarily reflects immediate or recent experiences of fear, tension, worry, and nervousness, and can be used to evaluate anxiety levels under stressful situations.

Items 21–40 make up the Trait Anxiety Inventory, which assesses people’s general, long-standing emotional experiences.

All items are scored on a 4-point Likert scale:

State Anxiety: 1 = Not at all, 2 = Somewhat, 3 = Moderately so, 4 = Very much so.

Trait Anxiety: 1 = Almost never, 2 = Sometimes, 3 = Often, 4 = Almost always.

Participants select the option that best reflects their experience. Separate total scores are calculated for the State and Trait subscales. The possible score range for each subscale is 20–80. A higher score indicates a higher level of anxiety in the corresponding domain.

Note: Items reflecting positive emotions are reverse scored. These are items 1, 2, 5, 8, 10, 11, 15, 16, 19, 20, 21, 23, 24, 26, 27, 30, 33, 34, 36, and 39.
