# Supplementary material for: Association between preoperative anxiety and ciprofol requirements in women undergoing surgical abortion: a prospective observational study
Source: Front Med (Lausanne). 2025 Dec 19;12:1706386. doi: 10.3389/fmed.2025.1706386 (PMC12757401; doi:10.3389/fmed.2025.1706386)
Supplement: Supplementary file 2 [file Data_Sheet_2.pdf]

Self-rating Anxiety Scale

NO.	Item	A little of the time	Some of the time	Good part of the time	Most of the time
1	I feel more nervous and anxious than usual				
2	I feel afraid for no reason at all				
3	I get upset easily or feel panicky				
4	I feel like I'm falling apart and going to pieces				
5*	I feel that everything is all right and nothing bad will happen				
6	My arms and legs shake and tremble				
7	I am bothered by headaches neck and back pain				
8	I feel weak and get tired easily				
9*	I feel calm and can sit still easily				
10	I can feel my heart beating fast				
11	I am bothered by dizzy spells				
12	I have fainting spells or feel like it				
13*	I can breathe in and out easily				
14	I get numbness and tingling in my fingers and toes				
15	I am bothered by stomach aches or indigestion				
16	I have to empty my bladder often				
17*	My hands are usually dry and warm				
18	My face gets hot and blushes				
19*	I fall asleep easily and get a good night's rest				
20	I have nightmares				

Scoring method: The SAS employs a 4-point scale to evaluate the frequency of symptoms: 1 = A little of the time; 2 = Some of the time; 3 = Good part of the time; 4 = Most of the time. Fifteen of the 20 items are negatively worded and are scored from 1 to 4 as described above. The remaining five items (5, 9, 13, 17, and 19), marked with an asterisk (*), are positively worded and scored in reverse order, from 4 to 1.

Analyzing indicators: The primary statistical metric in the SAS is the total score. Total score

is calculated by summing the scores of all 20 items, and the standardized score is derived by multiplying the total score by 1.25 and rounding to the nearest integer.
